# Use of the tumor-infiltrating CD8 to FOXP3 lymphocyte ratio in predicting treatment responses to combination therapy with pertuzumab, trastuzumab, and docetaxel for advanced HER2-positive breast cancer

**DOI:** 10.1186/s12967-018-1460-4

**Published:** 2018-04-03

**Authors:** Koji Takada, Shinichiro Kashiwagi, Wataru Goto, Yuka Asano, Katsuyuki Takahashi, Tsutomu Takashima, Shuhei Tomita, Masahiko Ohsawa, Kosei Hirakawa, Masaichi Ohira

**Affiliations:** 1 Department of Surgical Oncology, Osaka City University Graduate School of Medicine, 1-4-3 Asahi-machi, Abeno-ku, Osaka, 545-8585 Japan; 2 Department of Pharmacology, Osaka City University Graduate School of Medicine, 1-4-3 Asahi-machi, Abeno-ku, Osaka, 545-8585 Japan; 3 Department of Diagnostic Pathology, Osaka City University Graduate School of Medicine, 1-4-3 Asahi-machi, Abeno-ku, Osaka, 545-8585 Japan

**Keywords:** Tumor-infiltrating lymphocytes, Breast cancer, Pertuzumab, HER2, CD8/FOXP3 ratio

## Abstract

**Background:**

The trastuzumab, pertuzumab, and docetaxel (TPD) regimen is strongly recommended as a treatment option for first-line therapy for advanced human epidermal growth factor receptor (HER) 2-positive breast cancer. Monitoring the host microenvironments in cancer plays a significant role in predicting prognoses and curative effects. It is important to clarify the role of immune related gene expression in tumor-infiltrating lymphocytes in the tumor microenvironment. In this study, we evaluated the impact of chemotherapy with a TPD regimen, on immune micro environments in HER2-positive breast cancer using immune related proteins as indicators.

**Methods:**

The subjects consisted of 30 patients who received the TPD regimen. The expression levels of estrogen receptor, progesterone receptor, Ki67, CD8, forkhead box protein (FOXP) 3, programmed death (PD) 1, programmed death ligand (PD-L) 1, CD163, phosphatase and tensin homolog and lymphocyte activation gene 3 were evaluated in biopsy specimens, by immunostaining.

**Results:**

CD8^+^, CD8/FOXP3 ratio (CFR)^high^ and PD-L1^−^ group had significantly longer PFS than the CD8^−^, CFR^low^ and PDL1^+^ group (p = 0.045, log-rank) (p = 0.007, log-rank) (p = 0.040, log-rank), respectively. The CFR^high^ group had significantly better OS than the CFR^low^ group (p = 0.034, log-rank). In the univariate analysis, CD8^+^, CFR^high^ groups extended PFS significantly (p = 0.027, hazard ratio [HR] = 0.162) (p = 0.008, HR = 0.195), respectively. The receiver operating characteristic (ROC) analyses showed that the results for CFR [area under the curve (AUC): 0.708] were better than those for other factors (AUC: CD8 = 0.681, FOXP3 = 0.639, PD1 = 0.528, PD-L1 = 0.681).

**Conclusions:**

This study shows with the TPD regimen, a high CFR leads to a high ORR and long PFS in HER2-positive breast cancer. CFR, therefore, may be one of the important prognostic factors for this disease.

**Electronic supplementary material:**

The online version of this article (10.1186/s12967-018-1460-4) contains supplementary material, which is available to authorized users.

## Background

Human epidermal growth factor receptor (HER) 2, a tyrosine kinase transmembrane receptor, is associated with cellular growth and proliferation [1]. Overexpression of HER2 occurs in about 20% of breast cancers and leads to poor prognosis [2]. However, various monoclonal antibodies for HER2 protein, such as pertuzumab, are currently used in clinical treatment along with chemotherapy to improve prognosis. HER2 signaling plays an important role in HER2-positive breast cancer (HER2-BC) [3–5], the prognosis of which has been greatly improved by anti-HER2 therapy, mainly with trastuzumab [2, 6]. Large clinical trials have also shown that overall survival (OS) can be extended by treatment with pertuzumab [7–9], trastuzumab (a humanized monoclonal antibody), and trastuzumab emtansine (T-DM1; an antibody–drug conjugate) [10]. TPD regimen, consisting of trastuzumab, pertuzumab, and docetaxel, is the first choice of treatment for HER2-positive metastatic or recurrent breast cancer according to the National Comprehensive Cancer Network (NCCN) Guidelines Version 3.2015.

Monitoring the host microenvironments of cancer plays a significant role in predicting the prognoses and curative effects. Recently, there has an increase in reports that demonstrate the morphological evaluation of tumor-infiltrating lymphocytes (TILs) as well as its clinical implications in breast cancer. It is clear that TILs influence both the growth of various cancers and the action of anti-cancer drugs [11–14]. It is important to clarify the role of immune related genes in the tumor microenvironment. CD8^+^ cytotoxic T cells, lead to the death of tumor cells by apoptosis and improve prognosis [12]. In contrast, some cells, such as the regulatory T (Treg) cells and tumor associated macrophages (TAM), suppress anti-tumor immunity and promote proliferation of cancer [15–18]. We have previously reported the clinical validity and benefits of evaluating TILs for neoadjuvant chemotherapy (NAC). Chemotherapy with a TPD regimen (trastuzumab, pertuzumab, and docetaxel) has been established as the primary therapy for HER2-positive breast cancer and is garnering attention for its clinical outcomes and impact on cancer microenvironments. In this study, we evaluated the impact of chemotherapy with a TPD regimen, on immune micro environments in HER2-positive breast cancer using immune related proteins as indicators.

## Methods

### Patient background

This retrospective study was based on 30 patients with HER2-positive locally advanced or metastatic breast cancer, who received TPD regimen chemotherapy from September 2013 to November 2015 at the Osaka City University Hospital, Osaka, Japan.

The pathological diagnosis of breast cancer was made by core needle biopsy (CNB) or vacuum assisted biopsy (VAB), and the stage was decided by computed tomography (CT), ultrasonography (US), and bone scintigraphy. The median follow-up period for the assessment of progression-free survival (PFS) and OS was 357 days (range 42–1015 days) and 497 days (range 91–735 weeks), respectively.

TPD regimen consists of trastuzumab, pertuzumab, and docetaxel (DTX). The loading dose of trastuzumab was 8 mg/kg, and that of pertuzumab was 840 mg. The maintenance doses of trastuzumab and pertuzumab were 6 mg/kg and 420 mg, respectively every 3 weeks until disease progression. Six cycles of DTX were given at 75 mg/m^2^/cycle/3 weeks, but patients who were not healthy enough received 60 or 50 mg/m^2^ of DTX from the first cycle. If the doctors decided that continuing treatment with DTX was feasible, the patients underwent six additional cycles, but in the case of serious side effects, DTX was either reduced to 20–25% or was discontinued. At the end of the last cycle of DTX, all patients underwent imaging, and the outcomes were estimated in accordance with the response evaluation criteria in solid tumors (RECIST) criteria [19]. We defined the patients with objective response rate (ORR) as “Responders” and the others as “Non-responders”.

The morphology of the tumor was evaluated using conventional hematoxylin and eosin (HE) staining. The expression of estrogen receptor (ER), progesterone receptor (PgR), HER2 and Ki67 in the CNB or VAB specimens obtained prior to the start of chemotherapy with the TPD regimen, were evaluated using immunostaining. In cases of untreated metastatic and recurrent breast cancers, biopsy samples obtained for diagnosis and a surgical specimen of first-line treatment respectively were analyzed. The diagnosis was made by several experienced pathologists specializing in cancer. While OS was defined as the period from the start of treatment to death, PFS was the period from the start of treatment to the date of death or confirmation of progression disease (PD), whichever came earlier.

### Ethics statement

This study was conducted at Osaka City University Graduate School of Medicine, Osaka, Japan, according to the reporting recommendations for tumour marker prognostic studies (REMARK) guidelines and a retrospectively written, research, pathological evaluation, and statistical plan [20]. Informed consent was obtained from all patients. This research conformed to the provisions of the Declaration of Helsinki in 2013. The study protocol was approved by the Ethics Committee of the Osaka City University (#926).

### Immunohistochemistry

Immunohistochemistry studies were performed as described earlier [21]. Tumor specimens, which were fixed in 10% formaldehyde solution and embedded in paraffin, were sliced into 4-μm-thick sections and were mounted onto glass slides. The slides were deparaffinized in xylene and were incubated with 3% hydrogen peroxide in methanol for 15 min to block the endogenous peroxidase activity. Next, the specimens were heated for 10 min (105 °C) in an autoclave in the Target Retrieval Solution (Dako, Carpinteria, CA, USA). Primary monoclonal antibodies directed against ER (Dako, Cambridge, UK, clone 1D5, dilution 1:80;), PgR (Dako, clone PgR636, dilution 1:100; Dako), Ki-67 (Dako, clone MIB-1, dilution 1:100; Dako), HER2 (HercepTestTM; Dako), CD8 (Dako, clone C8/144B, dilution 1:150; Dako), forkhead box protein (FOXP) 3 (Abcam, Cambridge, UK, clone 236A/E7, dilution 1:150; Abcam, Cambridge, UK), programmed death (PD) 1 (Abcam, clone NAT105, dilution 1:200; Abcam), programmed death ligand (PD-L) 1 (Abcam, clone 28–8, dilution 1:150; Abcam), CD163 (Leica, Newcastle, UK, clone 10 D6, dilution 1:200; Leica, Newcastle, UK), phosphatase and tensin homolog (PTEN) (Dako, clone 6H2.1, dilution 1:150; Dako), lymphocyte activation gene (LAG) 3 (Abcam, clone 11E3, dilution 1:150; Abcam) were used (Additional file 1: Table S1). These sections were incubated with each antibody for 70 min at room temperature or overnight at 4 °C. Next, they were incubated for 10 min with horseradish peroxidase-conjugated anti-rabbit or anti-mouse Ig secondary antibodies [HISTOFINE (PO) TM kit; Nichirei, Tokyo, JAPAN]. Finally, the slides were treated with the streptavidin-peroxidase reagent and counterstained with Mayer’s hematoxylin.

### Immunohistochemical evaluation

The immunohistochemical staining was jointly scored by two breast pathologists (MOhs and STa), who were blinded to clinical information including treatments and outcomes. Based on the earlier study, samples with more than 1% staining were considered positive for ER and PgR, while those with less than 1% staining were counted as negative [22]. For Ki-67 expression, the cutoff was set at 14% based on the previous report [23]. TILs were evaluated in accordance with Salgado’s report [11]. TILs were defined as the infiltrating lymphocytes within the tumor stroma and were expressed as a proportion of the field investigated. The area of TILs in the stroma surrounding the stained cancer cells was quantitatively measured. Each field under 400× magnification [24, 25].

**Fig. 1:**
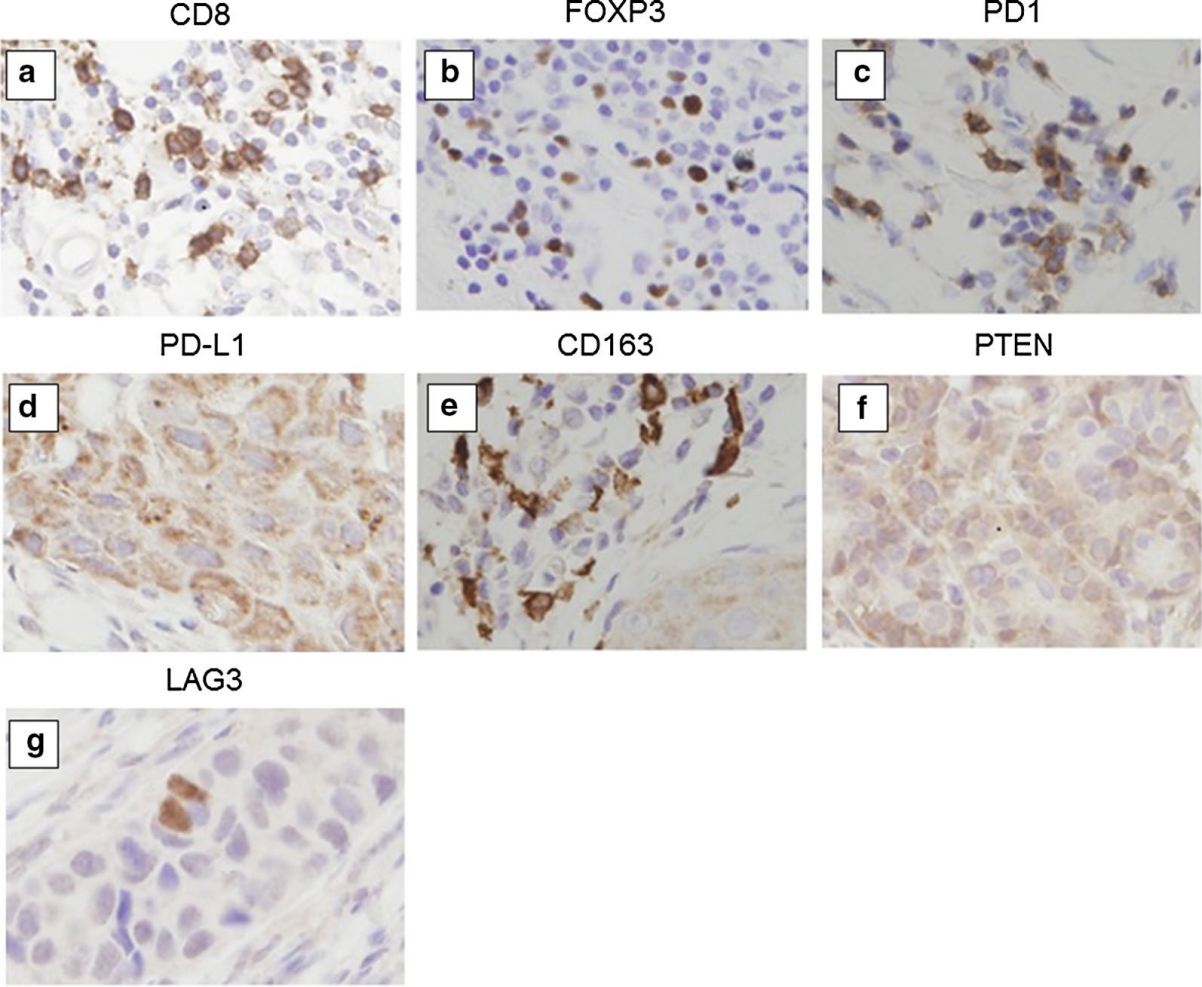
Evaluation of immune related proteins expression. These pictures were judged to be positive for expression (400-times magnification). Immunohistochemical staining using each monoclonal antibodies: **a** CD8, **b** FOXP3, **c** PD1, **d** PD-L1, **e** CD163, **f** PTEN, **g** LAG3

Based on previous studies, CD8, FOXP3, and CD163 expression were calculated using the average of stained TILs in the area that was maximally stained, viewed at 400× magnification [26, 27]. PD1 and LAG3 expressions were evaluated similarly, except that a 200× magnification was used for visualization. The CD8/FOXP3 ratio (CFR) was calculated using the CD8 and FOXP3 results. PD-L1 and PTEN were evaluated at three randomly selected areas on the basis of a previous analysis. PDL1 was evaluated as a percentage of cells stained with cell membranes at moderate or strong [28]. On the other hand, PTEN expression was scored using immunoreactive scores (IRS), which is the product of SI (staining intensity) and PP (percentage of positive cells). SI was scored as 0, 1, 2 and 3 for negative, weak, moderate and strong staining respectively. PP was scored as 0 (< 1%), 1 (1–10%), 2 (11–50%), 3 (51–80%) and 4 (> 80%) [29]. The cutoff value for each of the immune related proteins was calculated by the median value and previous reports [28]. As a result, the cutoff for CD8 was 40, FOXP3 was 20, CFR was 1.6, PD1 was 20, PD-L1 was 10, CD163 was 40, PTEN was 5, and LAG3 was 5 (Fig. 1) (Additional file 1: Table S1).

### Statistical analysis

Statistical analysis was performed using the JMP software (SAS, Tokyo, Japan). The relationship between each of immune related proteins and the ORR was examined by the Chi square test (or Fisher’s exact test when necessary). The Kaplan–Meier method and the log-rank test compared high and low expression about PFS and OS. The hazard ratio (HR) and 95% confidence intervals (CI) were calculated by the COX proportional hazards model. Univariate and multivariate analyses were performed by the Cox regression model and used in a backward stepwise method for variable selection in multivariate analysis. A *p* value of less than 0.05 was considered significant.

## Results

### Clinical characteristics

Thirty patients were given TPD regimen, and their clinical characteristics are listed in Table 1. The median age at the start of this regimen was 62 years (31–80 years). Eighteen (60%) and 20 (66.7%) patients were negative for ER and PgR respectively. Four patients (13.3%) were diagnosed with stage IIIC, 11 patients (36.7%) with stage IV, and 15 patients (50.0%) were diagnosed with a recurrence. The TPD regimen was used as the first line in 17 patients (56.7%). The dose of DTX was reduced in ten patients because of side effects. The median course of DTX was 6 cycles (1–9 cycle). The ORR of all patients was 80.0% (3 patients showed a complete response (CR), while 21 had a partial response). With the progression of HER2-positive breast cancer, 6 patients were moved to trastuzumab emtansine therapy, and 4 patients were given eribulin or capecitabine. While 4 patients died of breast cancer, 1 died of another disease, and yet another patient died of an unknown cause.

**Table 1:** Demographical data of 30 patients with TPD regimen for advanced HER2-positive breast cancer

Parameters (*n *= 30)	Number of patients (%)
Age (years old)	62 (31–80)
Degree of progress	
Locally advanced/visceral metastases	10 (33.3%)/20 (66.7%)
Stage	
Stage IIIC/stage IV/recurrence	4 (13.3%)/11 (36.7%)/15 (50.0%)
Site of metastases	
Lung/bone/liver/brain/lymph node/soft tissue	11 (36.7%)/12 (40.0%)/7 (23.3%)/3 (10.0%)/12 (40.0%)/11 (36.6%)
Treatment line	
First/other	17 (56.7%)/13 (43.3%)
ER	
Negative/positive	18 (60.0%)/12 (40.0%)
PgR	
Negative/positive	20 (66.7%)/10 (33.3%)
Ki67	
Negative/positive	16 (55.2%)/14 (44.8%)
CD8	
Negative/positive	21 (70.0%)/9 (30.0%)
FOXP3	
Negative/positive	15 (50.0%)/15 (50.0%)
CFR	
Low/high	15 (50.0%)/15 (50.0%)
PD1	
Negative/positive	14 (46.7%)/16 (53.3%)
PD-L1	
Negative/positive	19 (63.3%)/11 (36.7%)
CD163	
Negative/positive	15 (50.0%)/15 (50.0%)
PTEN	
Negative/positive	17 (56.7%)/13 (43.3%)
LAG3	
Negative/positive	20 (66.7%)/10 (33.3%)

TPD, trastuzumab, pertuzumab, docetaxel; HER2, human epidermal growth factor receptor 2; ER, estrogen receptor; PgR, progesterone receptor; FOXP3, forkhead box protein 3; CFR, CD8/FOXP3 ratio; PD1, programmed death 1; PD-L1, programmed death ligand-1; PTEN, phosphatase and tensin homolog; LAG3, lymphocyte activation gene 3

### Association of each immune-related protein with ORR, PFS, and OS

**Fig. 2:**
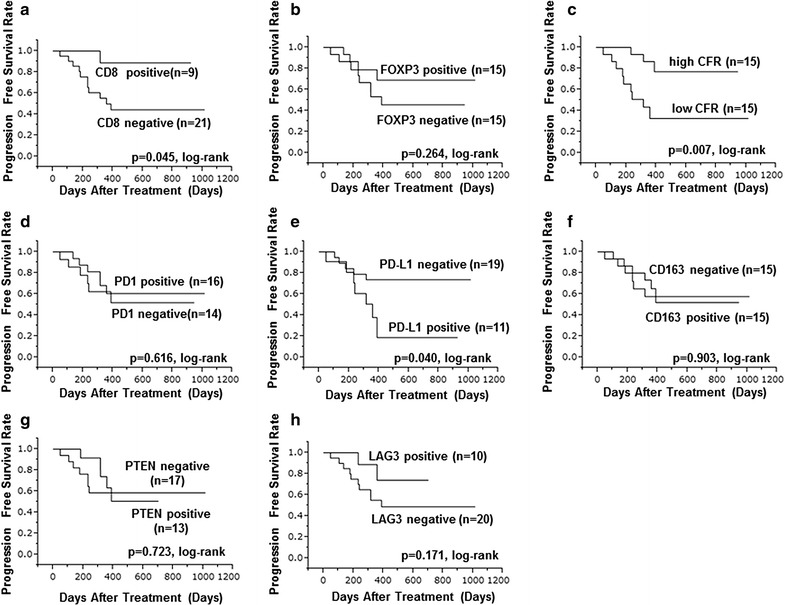
PFS with advanced HER2-positive breast cancer on immune related protein. Positive CD8, CFR^high^ group and negative PDL1 group had significantly longer PFS than negative CD8, CFR^low^ group and positive PDL1 group, respectively: **a** (p = 0.045, log-rank), **c** (p = 0.007, log-rank), **e** (p = 0.040, log-rank). Other immune related protein expression were not related to PFS: **b** FOXP3, **d** PD1, **f** CD163, **g** PTEN, **h** LAG3

**Fig. 3:**
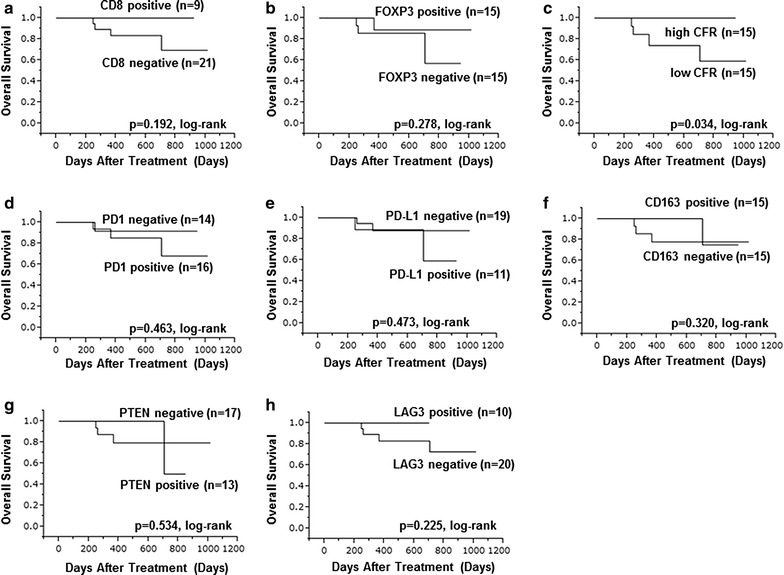
OS with advanced HER2-positive breast cancer on immune related protein. Positive or high groups had significantly better OS than negative or low group about CFR (**c**) (p = 0.034, log-rank). Other immune related protein expression were not related to OS: **a** CD8, **b** FOXP3, **d** PD1, **e** PD-L1, **f** CD163, **g** PTEN, **h** LAG3

When evaluating ORR, CFR^high^ group had significantly better ORR than CFR^low^ group (p = 0.013) (Table 2). There was no relationship between ORR and other immune related proteins. CD8^+^, CFR^high^ and PD-L1^−^ group had significantly longer PFS than negative CD8^−^, CFR^low^ and PDL1^+^ group (p = 0.045, log-rank) (p = 0.007, log-rank) (p = 0.040, log-rank), respectively (Fig. 2). The CFR^high^ groups also had significantly better OS than the CFR^low^ groups (p = 0.034, log-rank) (Fig. 3). Expression of other immune related proteins was not related to both PFS and OS.

**Table 2:** Correlation between immune related proteins and objective response rate (ORR) in 30 advanced HER2-positive breast cancer

Parameters	Responders (*n* = 25)	Non-responders (*n* = 5)	*p* value
Age at treatment			
≤ 62 vs > 62	12 (48.0%) vs 13 (52.0%)	3 (60.0%)/2 (40.0%)	0.638
Degree of progress			
Locally advanced vs visceral metastases	9 (36.0%)/16 (64.0%)	1 (20.0%)/4 (80.0%)	0.505
Stage			
IIIC or IV vs recurrence	12 (48.8%)/13 (52.0%)	3 (60.0%)/2 (40.0%)	0.638
ER			
Negative vs positive	16 (64.0%)/9 (36.0%)	2 (40.0%)/3 (60.0%)	0.334
PgR			
Negative vs positive	17 (68.0%)/8 (32.0%)	3 (60.0%)/2 (40.0%)	0.740
Ki67			
Negative vs positive	14 (56.0%)/11 (44.4%)	2 (40.0%)/3 (60.0%)	0.529
Treatment line			
First vs other	16 (64.0%)/9 (36.0%)	1 (20.0%)/4 (80.0%)	0.074
CD8			
Negative vs positive	16 (64.0%)/9 (36.0%)	5 (100.0%)/0 (0.0%)	0.116
FOXP3			
Negative vs positive	12 (48.8%)/13 (52.0%)	3 (60.0%)/2 (40.0%)	0.638
CFR			
Low/high	10 (40.0%)/15 (60.0%)	5 (100.0%)/0 (0.0%)	0.013
PD1			
Negative vs positive	11 (44.0%)/14 (56.0%)	3 (60.0%)/2 (40.0%)	0.529
PD-L1			
Negative vs positive	17 (68.0%)/8 (32.0%)	2 (40.0%)/3 (60.0%)	0.250
CD163			
Negative vs positive	12 (48.8%)/13 (52.0%)	3 (60.0%)/2 (40.0%)	0.638
PTEN			
Negative vs positive	13 (52.0%)/12 (48.8%)	4 (80.0%)/1 (20.0%)	0.264
LAG3			
Negative vs positive	16 (64.0%)/9 (36.0%)	4 (80.0%)/1 (20.0%)	0.505

ORR, objective response rate; HER2, human epidermal growth factor receptor 2; ER, estrogen receptor; PgR, progesterone receptor; FOXP3, forkhead box protein 3; CFR, CD8/FOXP3 ratio; PD1, programmed death 1; PD-L1, programmed death ligand-1; PTEN, phosphatase and tensin homolog; LAG3, lymphocyte activation gene 3

**Fig. 4:**
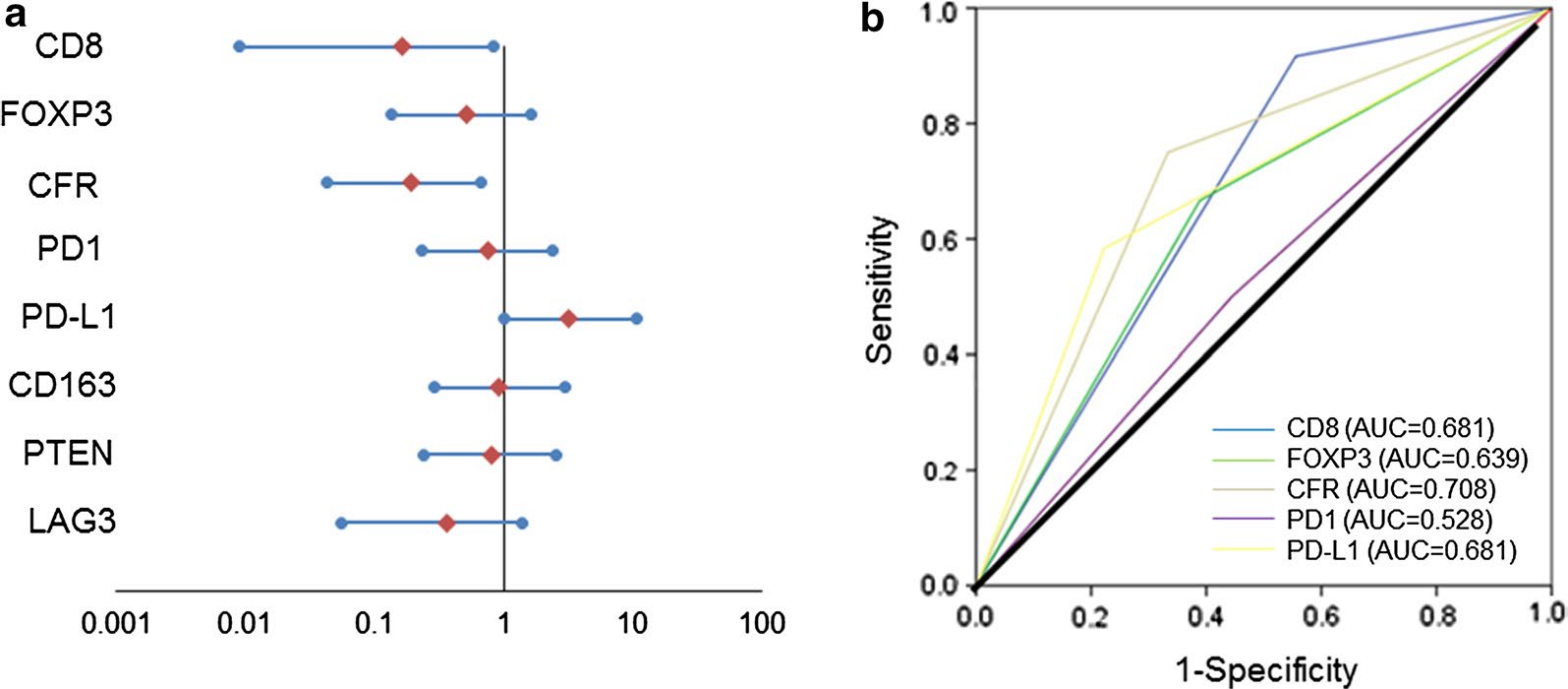
Forest plots and on ROC analyses. **a** In univariate analysis, positive CD8, CFR^high^ group made a significant contribution to extending PFS in patients with advanced HER2-positive breast cancer, respectively (p = 0.027, HR = 0.162) (p = 0.008, HR = 0.195). **b** ROC analyses showed that, for advanced HER2-positive breast cancer patients, the results for the CFR (AUC: CFR = 0.708) were better than those for the other factors (AUC: CD8 = 0.681, FOXP3 = 0.639, PD1 = 0.528, PD-L1 = 0.681)

In a univariate analysis, CD8^+^, CFR^high^ group made a significant contribution to extending PFS in patients with advanced HER2-positive breast cancer (p = 0.027, hazard ratio [HR] = 0.162) (p = 0.008, HR = 0.195), respectively (Fig. 4a). However, a multivariate analysis revealed that CD8^+^, CFR^high^ and PD-L1^−^ group was not an independent factor (p = 0.292, HR = 0.333) (p = 0.149, HR = 0.336) (p = 0.582, HR = 1.441) (Table 3).

**Table 3:** Univariate and multivariate analysis with respect to progression free survival (PFS) in 30 advanced HER2-positive breast cancer

Parameters	Univariate			Multivariate		
	Hazard ratio	95% CI	*p* value	Hazard ratio	95% CI	*p* value
Age at treatment						
≤ 62 vs > 62	1.271	0.395–4.096	0.680			
Degree of progress						
Locally advanced vs visceral metastases	0.934	0.293–3.505	0.912			
Stage						
IIIC or IV vs recurrence	1.260	0.402–4.263	0.692			
ER						
Negative vs positive	1.581	0.494–5.063	0.431			
PgR						
Negative vs positive	0.925	0.246–2.948	0.898			
Ki67						
Negative vs positive	1.066	0.333–3.410	0.912			
Treatment line						
First vs other	1.449	0.452–4.643	0.522			
CD8						
Negative vs positive	0.162	0.009–0.837	0.027	0.333	0.017–2.349	0.292
FOXP3						
Negative vs positive	0.513	0.137–1.632	0.263			
CFR						
Low/high	0.195	0.043–0.664	0.008	0.336	0.062–1.455	0.149
PD1						
Negative vs positive	0.751	0.234–2.404	0.620			
PD-L1						
Negative vs positive	3.147	0.994–10.754	0.051	1.441	0.400–5.768	0.582
CD163						
Negative vs positive	0.932	0.291–2.992	0.904			
PTEN						
Negative vs positive	0.814	0.240–2.556	0.725			
LAG3						
Negative vs positive	0.364	0.056–1.386	0.149			

PFS, progression free survival; HER2, human epidermal growth factor receptor 2; CI, confidence intervals; ER, estrogen receptor; PgR, progesterone receptor; FOXP3, forkhead box protein 3; CFR, CD8/FOXP3 ratio; PD1, programmed death 1; PD-L1, programmed death ligand-1; PTEN, phosphatase and tensin homolog; LAG3, lymphocyte activation gene 3

Receiver operating characteristic (ROC) analyses showed that, for advanced HER2-positive breast cancer patients, the CFR results [area under the curve (AUC): 0.708] were better than those for the other factors (AUC: CD8 = 0.681, FOXP3 = 0.639, PD1 = 0.528, PD-L1 = 0.681) (Fig. 4b).

## Discussion

The tumor microenvironment plays an important role in cancer treatments. TILs are known to affect the tumor growth and the antitumor treatments in various cancers [30–33]. Among the TILs, cytotoxic CD8^+^ cells play an important role in antitumor effect [31]. Conversely, there are some cells and proteins that promote tumor proliferation or suppress the antitumor effects of CD8^+^ T cells. Treg cells which express FOXP3, belong to this class and inhibit the CD8^+^ T-cells [30]. As the proportion of FOXP3 increases, it interferes with the activity of CD8^+^ T cells, even in the presence of high levels of CD8, and therefore, CFR was used in some studies.

PD1, a transmembrane protein expressed on T cells, B cells and natural killer T cells, regulates immune tolerance and autoimmunity [31]. PD1 has two ligands PDL1 and PDL2 [30]. While PDL2 is expressed on dendritic cells and macrophages, PDL1 is expressed not only on resting T cells, B cells, dendritic cells, and macrophages but also on a number of different cancer cells [30, 32]. Due to its inhibitory effect on the antitumoral T cell-mediated immunity, the PD1/PDL1 pathway is a poor prognostic indicator in various cancers [32–36]. Studies have shown that the inhibition of PD1/PDL1 pathway enhances the antibody-dependent cell-mediated cytotoxicity (ADCC) of natural killer cells and induces apoptosis in tumor cells [37–39]. Furthermore, Paul et al. have demonstrated that the therapeutic effects of targeting PD1 are related to the CD8^+^ T cells in invasive cancer prior to therapy [40]. CD163, a single-chain transmembrane protein expressed in mature macrophages and monocytes, is regarded as a specific marker for M2 macrophages [41]. Macrophages are divided into a classically activated phenotype (M1) and an alternatively activated phenotype (M2). TAM are M2 macrophages which are in and around the tumor [17]. Therefore, in this study, we studied CD163 expression by immunostaining to identify TAM. TAM produce a variety of immunosuppressive molecules and promote angiogenesis and tissue remodeling [42]. These features accelerate tumor growth and contribute to poor prognosis in most cancers, including breast cancer [17, 41]. LAG3, also known as CD223, is expressed on natural killer cells, B cells, and dendritic cells. It binds to MHC class II [43]. Recent studies have shown LAG3 is expressed on CD8^+^ T cells and Treg cells in TILs, and it inhibits the activity of CD8^+^ T cells [43, 44]. Therefore, new drugs that block the interaction between LAG3 and MHC class II are being clinically studied.

Although we have described the relationship between immune related proteins and chemotherapy, there are very few reports that study molecular targeted drugs. We, therefore, investigated the role of the immune system in a typical chemotherapy including molecular targeted drugs, such as TPD therapy. Trastuzumab has been used as a monoclonal antibody for HER2 protein before pertuzumab. Trastuzumab emtansine combination has been used in the clinic, but most patients develop resistance to trastuzumab within a year [45]. Reduced PTEN expression has been reported to be the most likely cause of resistance [46]. PTEN antagonizes phosphatidylinositol 3-kinase function and regulates Akt activities. We suspect that a similar mechanism might be in play during the TPD regimen, but it is yet to be confirmed.

As a limitation of this study, protein expression was only retrospectively assessed by immunostaining. In order to show the relationship between tumor and its immune microenvironment, it is necessary to evaluate at the gene level and prove it with vitro. However, CFR or PDL1 expression was significantly correlated with PFS, as seen from the univariate analysis. Moreover, a higher CFR was correlated with a better ORR and a longer PFS in the multivariate analyses. These results suggest that the good immune tumor microenvironment enhance the antitumor effects of TPD therapy. It was suggested that CFR is the most sensitive indicator about the immune tumor microenvironment monitoring in TPD regimen chemotherapy.

## Conclusions

This study shows that high CFR leads to high ORR and long PFS under TPD regimen in HER2-positive breast cancer and that CFR may become one of the prognostic factors. We present the importance of cancer microenvironment in tumor immunology among anti-tumor treatment and reaffirmed the necessity of examining improvement methods in tumor immunology for patients diagnosed with poor prognosis including low CFR group in the future.

## Additional file

**Additional file 1: Table S1.** Primary antibodies for immunohistochemistry and immunohistochemical evaluation.

## Abbreviations

TPD: trastuzumab, pertuzumab, docetaxel
HER2: human epidermal growth factor receptor 2
TILs: tumor-infiltrating lymphocytes
ER: estrogen receptor
PgR: progesterone receptor
FOXP3: forkhead box protein 3
PD-1: programmed death-1
PD-L1: programmed death-ligand 1
PTEN: phosphatase and tensin homolog
LAG3: lymphocyte activation gene 3
CFR: CD8/FOXP3 ratio
HR: hazard ratio
ROC: receiver operating characteristic
AUC: area under the curve
BC: breast cancer
OS: overall survival
T-DM1: trastuzumab emtansine
NCCN: National Comprehensive Cancer Network
Treg: regulatory T
TAM: tumor associated macrophages
NAC: neoadjuvant chemotherapy
CNB: core needle biopsy
VAB: vacuum assisted biopsy
CT: computed tomography
US: ultrasonography
PFS: progression-free survival
DTX: docetaxel
RECIST: response evaluation criteria in solid tumors
ORR: objective response rate
HE: hematoxylin and eosin
PD: progression disease
REMARK: reporting recommendations for tumour marker prognostic studies
IRS: immunoreactive scores
CI: confidence intervals
ADCC: antibody-dependent cell-mediated cytotoxicity
