# Supplementary material for: Use of the tumor-infiltrating CD8 to FOXP3 lymphocyte ratio in predicting treatment responses to combination therapy with pertuzumab, trastuzumab, and docetaxel for advanced HER2-positive breast cancer
Source: J Transl Med. 2018 Apr 3;16:86. doi: 10.1186/s12967-018-1460-4 (PMC5883877; doi:10.1186/s12967-018-1460-4)
Supplement: Supplementary file 1 — Additional file 1: Table S1. Primary antibodies for immunohistochemistry and immunohistochemical evaluation. [file 12967_2018_1460_MOESM1_ESM.docx]

**Additional file 1: Table S1 Primary antibodies for immunohistochemistry and immunohistochemical evaluation.**

| Antibody | Clone | Dilution | Evaluation part | Evaluation method | Cut-off (%) |
| --- | --- | --- | --- | --- | --- |
| ER | clone 1D5 | 1:80 | Nucleus | Proportion | 1 |
| PgR | clone PgR636 | 1:100 | Nucleus | Proportion | 1 |
| Ki67 | clone MIB-1 | 1:100 | Nucleus | Proportion | 14 |
| CD8 | clone C8/144B | 1:150 | TILs | Number in view | 40 |
| FOXP3 | clone 236A/E7 | 1:150 | TILs | Number in view | 20 |
| PD1 | clone NAT105 | 1:200 | TILs | Number in view | 20 |
| PD-L1 | clone 28-8 | 1:150 | Membrane | Proportion | 10 |
| CD163 | clone 10D6 | 1:200 | TILs | Number in view | 40 |
| PTEN | clone 6H2.1 | 1:150 | Cytoplasm | Immunoreactive scores | 5 |
| LAG3 | clone 11E3 | 1:150 | TILs | Number in view | 5 |

ER, Estrogen receptor. PgR, Progesterone receptor. FOXP3, forkhead box protein 3. PD1, programmed death 1. PD-L1, programmed death ligand-1.PTEN, phosphatase and tensin homolog. LAG3, lymphocyte activation gene 3. TILs, tumor-infiltrating lymphocytes.
